# Neuronal Correlates of a Virtual-Reality-Based Passive Sensory P300 Network

**DOI:** 10.1371/journal.pone.0112228

**Published:** 2014-11-17

**Authors:** Chun-Chuan Chen, Kai-Syun Syue, Kai-Chiun Li, Shih-Ching Yeh

**Affiliations:** 1 Graduate Institute of Biomedical Engineering, National Central University, Jhongli city, Taoyuan County, Taiwan; 2 Department of Computer Science and Information Engineering, National Central University, Jhongli city, Taoyuan County, Taiwan; University Of Cambridge, United Kingdom

## Abstract

P300, a positive event-related potential (ERP) evoked at around 300 ms after stimulus, can be elicited using an active or passive oddball paradigm. Active P300 requires a person’s intentional response, whereas passive P300 does not require an intentional response. Passive P300 has been used in incommunicative patients for consciousness detection and brain computer interface. Active and passive P300 differ in amplitude, but not in latency or scalp distribution. However, no study has addressed the mechanism underlying the production of passive P300. In particular, it remains unclear whether the passive P300 shares an identical active P300 generating network architecture when no response is required. This study aims to explore the hierarchical network of passive sensory P300 production using dynamic causal modelling (DCM) for ERP and a novel virtual reality (VR)-based passive oddball paradigm. Moreover, we investigated the causal relationship of this passive P300 network and the changes in connection strength to address the possible functional roles. A classical ERP analysis was performed to verify that the proposed VR-based game can reliably elicit passive P300. The DCM results suggested that the passive and active P300 share the same parietal-frontal neural network for attentional control and, underlying the passive network, the feed-forward modulation is stronger than the feed-back one. The functional role of this forward modulation may indicate the delivery of sensory information, automatic detection of differences, and stimulus-driven attentional processes involved in performing this passive task. To our best knowledge, this is the first study to address the passive P300 network. The results of this study may provide a reference for future clinical studies on addressing the network alternations under pathological states of incommunicative patients. However, caution is required when comparing patients’ analytic results with this study. For example, the task presented here is not applicable to incommunicative patients.

## Introduction

Neuronal activities as measured with electroencephalogram (EEG)/MEG are the direct window for studying the living brain at work. Specifically, P300, a positive event-related potential (ERP) evoked at around 300 ms after stimulus [1] has been investigated intensively and thought to reflect the higher level cognitive processes like selective attention and memory updating [2], [3]. P300 can be elicited reliably in an oddball paradigm using a variety of stimuli, such as visual, auditory or sensory stimuli. There are two types of P300: active and passive P300. Active P300 requires the subjects’ attention to response, while passive P300 requires no intentional response [2]. Active P300 has been successfully applied to discriminate the abnormality from the healthy based on its amplitude and latency [3]. For example, P300 with prolonged latencies and markedly lower amplitudes are characteristic of patients with Alzheimer’s disease [4], [5]. Because of the medical significance of P300 activity, numerous studies have investigated the neuronal origin and the underlying mechanisms involved in generating active P300, although conclusions have been inconsistent. For instance, Downar et al. have identified the neuroanatomical correlates underlying the detection of changes in the sensory environment using event-related functional magnetic-resonance imaging (fMRI) and a modified oddball paradigm [6]. They concluded that a distributed, right-lateralized network-comprising temporoparietal junction (TPJ), inferior frontal gyrus (IFG), insula and left cingulate and supplementary motor areas (CMA/SMA) as well as the primary sensory cortex (SI)–responds to changes in multiple sensory modalities and the subsequent involuntary attention shift. The aforementioned areas correspond closely to lesions in hemineglect patients and are considered as the underlying mechanism of the P300 production. Crottaz-Herbette and Menon examined the attentional control network by using simultaneous fMRI and EEG data recorded while performing auditory and visual oddball attention tasks [7]. They showed that the anterior cingulate cortex (ACC), left premotor area (PMA), inferior parietal lobule (IPL), and the primary sensory areas formed a network underlying the generation of P300 and that the ACC plays a crucial role in the top-down modulation of sensory processing. In 2005, Huang et al., employed MEG to study the engagement of the distributed parietal-frontal network in a median-nerve oddball paradigm [8]. They found that the same parietal-frontal neuronal network activated by both visual and auditory changes can also be activated by somatosensory stimulation. Recently, Brazdil et al. further studied the effective connectivity between right IPL, ACC and lateral prefrontal cortex (PFC) using fMRI and Dynamic Causal Modelling (DCM) during a visual oddball task [9]. They concluded that bidirectional coupling occurs between the frontal and parietal regions and that the ACC exerts influence over PFC mediating the top-down attentional control. In summary, the frontal, parietal and temporal cortices, and primary sensory cortex are associated with the generation of active P300, though the engagement of brain areas is subjective to various stimuli and measurement modalities (for a more detail review, see Appendix S1). As for passive P300, numerous studies have shown that the passive oddball paradigm is adequately sensitive for probing the conscious state of incommunicative brain trauma or stroke patients [10], [11], [12]. Furthermore, passive P300 waves have been used in brain-computer interfaces (BCIs) because of its advantages of no training required and the results were significant [13], [14]. Although active and passive P300 differ only in the amplitude but not in the latency and scalp distribution [15], [16], [17], it was suggested that different neuronal pathways and networks may be involved in the active and passive conditions. However, to our best knowledge, the mechanism underlying the passive P300 production has not been addressed. In particular, it remains unclear whether the passive conditions share the active P300-generating network architecture when no response is required.

Virtual reality (VR) is a computer-based environment that provides the users an immersive experience of a synthetic world. Since the development of VR, researchers have been applying VR technologies continually in various medical contexts, such as diagnosis, presurgical rehearsal and planning, as well as stroke rehabilitation [18]. VR provides a simulated environment and features controllable parameters, thereby facilitating the systemic testing of human functions in both healthy and diseased states [19], [20]. Thus, we designed a VR-based passive sensory oddball task to elicit the passive P300 and investigated the underlying neuronal network by applying for ERP [21], [22]. A passive sensory oddball task was selected for this study because sensory P300 can be evoked reliably and is suitable for BCI applications and consciousness probing. Traditional passive sensory oddball paradigms either apply only sensation to skin (ex. vibration or painless electrical stimulation), or use median nerve electrical stimulation that induces both sensory and motor responses at the same time. The common difficulty for a passive task is that there is no way to assure the attentive of the subjects. Lack of attention may decrease largely the amplitude of P300 and lead to a non-significant result [23]. Moreover, the hand movement produced by median nerve stimulation may cause a confound when investigating the underlying neuronal network since there exists a movement-related top-down modulation during a simple movement task [24]. Specifically, it has not been tested whether the backward modulation, if there has any, is engaged under a passive condition, or just is a reflection of movement-related top-down control. Therefore, in this study, we take the advantage of VR to design a task that can keep the subjects’ attention and is still able to separate the movements from sensory change detection. We expect that, this task will evoke the somatosensory evoked potentials (SEPs) of P50, N80, and P200 [25] during the standard events, as well as passive P300 during the rare events. Based on this expectation, we aim to investigate this passive sensory P300 network by DCM for ERP. Importantly, the causal relationship of this passive P300 network and the changes in connection strength will be examined to address the possible functional roles. This paper is organized as follows; The subsequent section outlines the experimental protocol design and details the plausible dynamic causal models. The final section presents the results and the implications of the findings.

## Materials and Methods

Written consent was obtained from all subjects for the experiment with a protocol approved by the institutional review board of Taipei Veterans General Hospital.

### Subjects and Task

Ten healthy, right-handed male college students (22–29 years of age) were recruited for this study. Written consent was obtained from all subjects for the experiment with a protocol approved by the institutional review board of Taipei Veterans General Hospital, in accordance with the Declaration of Helsinki. The subjects sat comfortably wearing goggles, through which they viewed a virtual robot pitching a baseball toward them at 4±0.5 second intervals (mean interval between trials = 4 seconds). The angle and speed of the ball varied between trials to prevent the subjects from anticipating the characteristics of the next pitch. They were instructed to catch the virtual ball by using a game controller held in their right hand, and no further instruction was given regarding the actions to be taken after catching it. This VR-based ball-catching task was performed under 2 conditions (standard and rare) characterized by different occurrence frequencies. The standard condition (480 trials; 80% occurrence) was designed to mimic the real-world conditions of a person catching a ball. When the subjects successfully caught the virtual ball, haptic stimuli (i.e. the sensory force feedback) were delivered through the game controller. The rare condition (120 trials; 20% occurrence) was designed to elicit passive P300 by removing the haptic stimuli, even when the subjects successfully caught the ball. In other words, a passive P300 will be produced by the occasional lack of haptic feedback, i.e. the rare conditions, while the SEPs will be evoked during the standard events. As the haptic feedback came after the movement finished (i.e. the ball has been caught), this task allows us to bypass the possible movement-related top-down modulation that may cause a confound [24].

### EEG Acquisition and Processing

Thirty-channel EEG data (10–20 system montage; QuickAmp amplifier by Brain Products), referred to linked earlobes with a forehead ground, were recorded at a sampling rate of 250 Hz during the ball catch task. Waveforms were further re-referenced on-line by common average across all channels. The position of the EEG electrodes was measured using an optical 3D electrode digitizer system (Xensor) before starting the EEG-recordings. The locations of the subject-specific channels will be used in DCM for co-registration of the EEG coordinates with the canonical template MRI images in SPM. The data were epoched offline by using SPM8 (Wellcome Trust Centre for Neuroimaging, available at: http://www.fil.ion.ucl.ac.uk/spm/), with a peristimulus window from –500 to 1000 ms, where Time 0 denoted the moment the ball was caught. Poorly performed trials (i.e. when the subjects failed to catch the virtual ball) were excluded from further analysis, resulting in 467±26 and 118±6 trials for standard and rare events, respectively. EOG contamination (i.e. EEG amplitude >100 mV) was removed from the epochs by using a fully automated correction method [26] and these EOG-free trials were divided into standard and rare groups according to their occurrences. The epochs of both groups were lowpass filtered (cutoff frequency = 30 Hz), baseline corrected (–2000 ms) and averaged across trials. The mean ERPs in both groups were first examined phenomenonally to identify the SEPs and P300. Subsequently, the ERPs entered DCM of ERPs as the observations that the DCM models are trying to explain.

### Model specification for DCM of ERP

**Figure 1:**
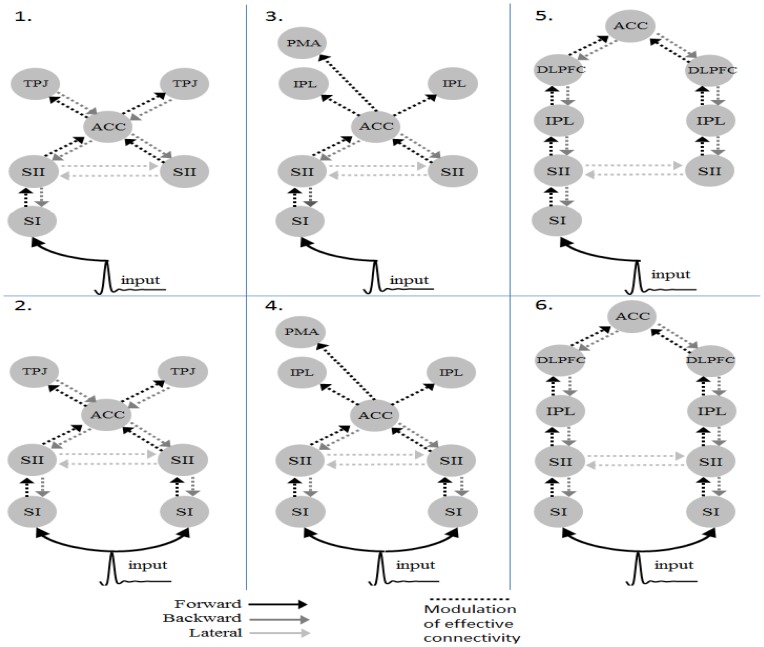
The architectures of the plausible model pairs. (SI : primary sensory area; SII : secondary sensor area; ACC : Anterior cingulate cortex; TPJ: Temporoparietal junction; IPL : Inferior parietal lobule; PMA : Premotor area;DLPFC: Dorsolateral prefrontal cortex).

In DCM for ERP analysis, we use only neuronal responses recorded between 0 and 900 ms, because these signals capture the entire stimulus duration and cortical responses for P300 as well as exclude the movement-related responses. To reduce the number of potential model combinations, we applied the following 2-step strategy. **Firstly**, we specified 3 plausible model pairs (Figure 1) to identify the most likely model hierarchy based on 3 previous studies discussed in the Introduction section [6], [7], [8]. Specifically, we addressed the following 2 factors: (1) whether the ACC is at the top of the passive network hierarchy; and (2) whether bilateral inputs are essential to generate activities in this sensory cognitive network as this task involves a unilateral stimulus only. These models share common structures, including SI ([-34 -31 56; 37-30 54]), secondary somatosensory areas (SII, [-59 -28 24; 50-28 25]) and ACC ([1 4 29]) [6], yet they differed in some higher areas and connections. Model 1 includes the bilateral TPJ ([-54 -48 10; 54-42 13]), which may be involved in detecting changes in the sensory environment [6]. Model 3 has bilateral IPL [-40 -38 56; 46-26 32] and left PMA [-32 -16 -64] for mediating the attentional control [7]. It should be noted that, in Crottaz-Herbette and Menon’s study, SMA has been suggested as a node in this distributed network. Because the spatial resolution of our EEG system is not good enough to distinguish the activity of SMA from that of ACC, for simplicity, we used ACC here to represent the activities of ACC/SMA as the report of Downar et al. [6] shows. Model 5 specifies a distributed parietal-frontal network that is activated during the sensory oddball task using MEG data. This network comprises bilateral DLPFC ([-34 25 29; 37 23 30]) and IPL ([-37 50 46; 46 46 41]) [8]. Model 2, 4 and 6 differ from Model 1, 3 and 5 in the present of the sensory input to the right SI (Figure 1). These source locations in the models (in Talairach coordinate) were taken from the cited studies that motivated the models during an active oddball task. The task-related modulation in these networks was pre-assumed to be reciprocal in all connections. **Secondly**, once we have established the most likely network, we then further examine where the task specific modulation takes place. We altered the task-related modulation as forward (F), backward (B) or lateral (L), thereby constructing 5 additional models (denoted as F, B, FB, FL and BL). In addition, we grouped the 6 models into 3 families: F (F+FL), B (B+BL) and FB (FB+FBL) to compare the model families to draw inferences on the importance of the directionality of the modulatory connection, independent of any uncertainty associated with the model structure [27]. Note that we also used a recently validated third-party software package [28] to accelerate the computation of DCM for ERPs.

### Statistical Tests and Bayesian Model Selection

A repeated measures analysis of variance (rmANOVA) was applied to test the experimental factors (conditions x electrode locations; 230) given the peak amplitude of P300 over all 10 subjects. The window of P300 was set to be between +250 to +800 ms after the ball was caught. Post hoc tests (Bonferroni-Dunn correction) were performed to assess differences in peak amplitude among factors. Because the elicitation of SEPs is most reflective at primary somatosensory cortex, we used the paired *t*-test to examine the peak amplitudes of SEPs (P50, N80 and P200) at C3 within a window from 0 to 250 ms, of the standard and rare conditions to ensure that the haptic stimulus were delivered successfully in the standard condition. Results were considered statistically significant when P<.05 after correction for multiple comparisons (Bonferroni-Dunn correction).

For DCM analysis, we used Bayesian model selection (BMS) to identify the best models among the models being tested at the individual level under the fixed effects assumption (FFX) [29]. At the group level, we applied the random effects (RFX) assumption to accommodate inter-individual variability in the structure of models or functional architectures that gave rise to individual-specific brain activity while performing the task [27], [30], [31]. After identifying the wining model by BMS, we tested the modulatory effect of the experimental manipulation by performing a *t* test to identify significant modulatory network parameters.

## Results

### Behavioral Data and SEPs

**Figure 2:**
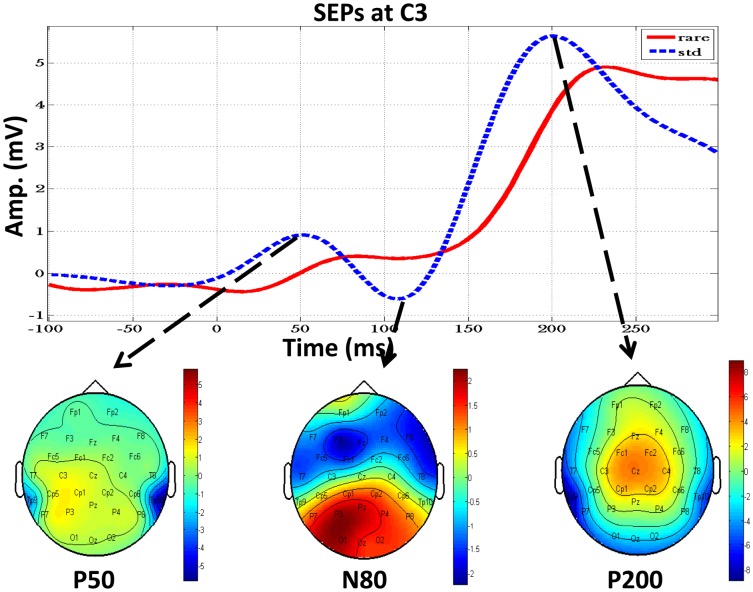
The time courses of the mean SEPs under standard (dash line) and rare (solid line) conditions at C3 and the mean topographic maps at the individual peak of P50, N80 and P200.

The behavioral data revealed that the task performance of all subjects was consistent (mean miss rate = 3.5%, mean reaction time = 939.9±21.7 ms), indicating that all of the subjects were attentive. The SEPs (i.e. P50, N80, and P200) were examined to ensure that (1) the haptic stimulus was successfully delivered in the standard condition and (2) the absence of this stimulus elicited P300 activity. Figure 2 shows the time courses of the mean SEPs of all subjects under both standard and rare conditions at electrode C3 and the corresponding topographic maps at the individual peak of P50, N80 and P200 under the standard condition. The peak amplitudes of SEPs elicited under the standard condition were higher than those elicited under the rare condition, although the differences were non-significant for P50 (*P* = .2), N100 (*P* = .06), and P200 (*P* = .07). The scalp topographies of the P50 and N80 components were more prominent over the regions contralateral to the stimulation, whereas the P200 peak amplitude exhibited a centroparietal-dominant scalp distribution with maximal amplitudes at electrode Cz. The *t* test results of the SEPs amplitudes at electrode C3 and C4 under the standard condition confirmed that there is a significant lateralization effect (P<0.004 for P50, P<0.031 for N100 and P<0.044 for P200). These results are in line with the previous study [25].

### P300

**Figure 3:**
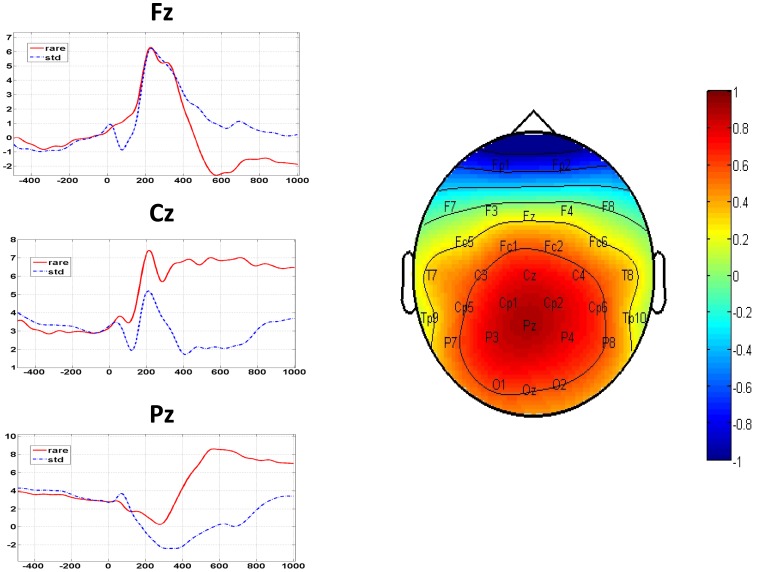
The time courses of the ERP at Fz, Cz and Pz averaged across subjects (left) and the mean topographic map at the individual peak of P300, normalized to the individual-specific maximum and minimum (right).

**Figure 4:**
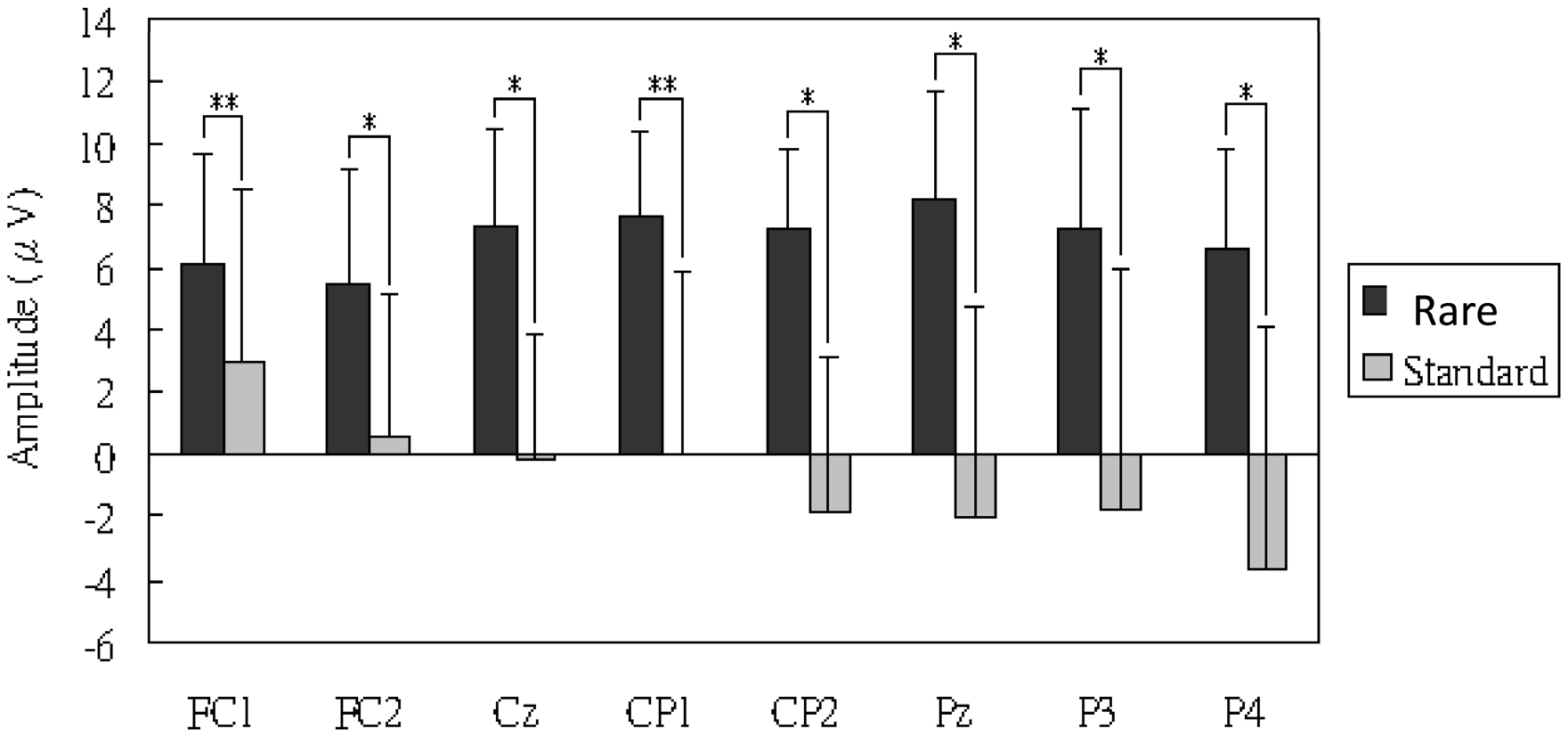
The statistic result revealed by the Post-hoc paired *t*-test on the P300 amplitude. (*: P<0.001, **: P<0.05).

Figure 3 shows the time courses of the mean ERPs at Fz, Cz and Pz (averaged across subjects; left side of the figure) and the mean topographic map at the P300 peak amplitudes for each subject (normalized to the individual-specific maximum and minimum; right side of the figure). The central-parietal areas (Cz and Pz) exhibited the greatest difference in mean amplitude between the 2 conditions but the difference was non-significant at Fz. The rmANOVA results of the P300 peak amplitudes of all 30 channels confirmed a significant effect on the condition (F[1.00, 9.00] = 6.638, *P* = .0299) and location (F[1.27, 11.44] = 4.781, *P* = .0435) as well as their interaction (condition × location; F[2.61, 23.46] = 11.411, *P* = .0001). Post-hoc paired *t*-test after correction for multiple comparisons further identified 8 channels, including FC1 (p = 0.0011), FC2 (p = 0.0003), Cz (p = 0.0001), CP1 (p = 0.0013), CP2 (p = 0.0001), Pz (p = 0.0001), P3 (p = 0.0005) and P4 (p = 0.0002) (Figure 4), supporting that the P300 component was elicited in this task. Table 1 summarizes the mean peak amplitudes and latencies across all subjects at the above 8 electrodes.

**Table 1:** The mean P300 peak amplitude and latency under the rare condition.

Electrode	Amplitude (µV)(mean± standard deviation)	Latency (ms)(mean ± standard deviation)
FC1	6.09±3.50	403.67±215.88
FC2	5.46±3.78	408.67±171.38
Cz	7.34±3.19	442.33±143.58
CP1	7.65±2.72	544.67±119.01
CP2	7.28±2.51	521.67±127.54
Pz	8.15±3.49	549.33±108.47
P3	7.28±3.84	617.00±125.89
P4	6.59±3.25	563.33±129.75

### Inferences on Model Space

**Figure 5:**
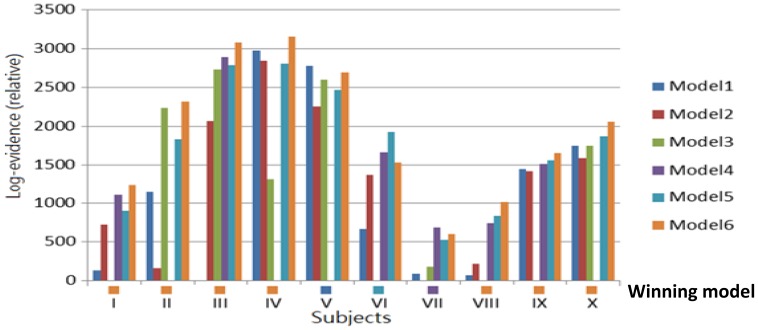
The result of BMS at the single subject level under FFX.

**Figure 6:**
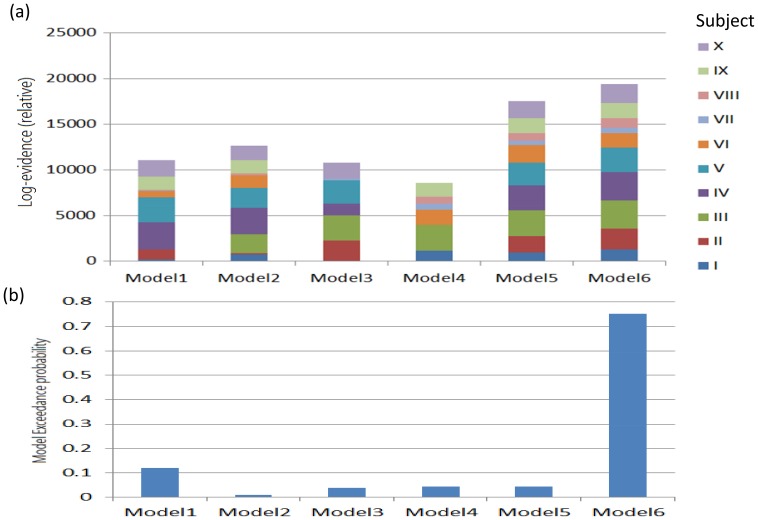
BMS results at the group level under FFX (a) and RFX (b) both confirmed that Model 6 is the most likely model hierarchy among tested models.

**Figure 7:**
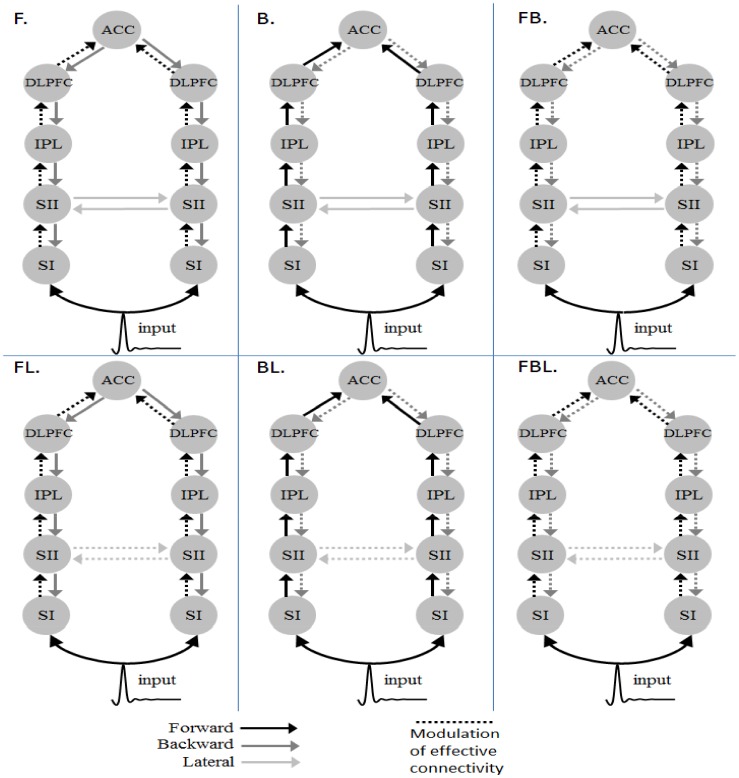
The models for testing the mechanism of the task-related modulation.

**Figure 8:**
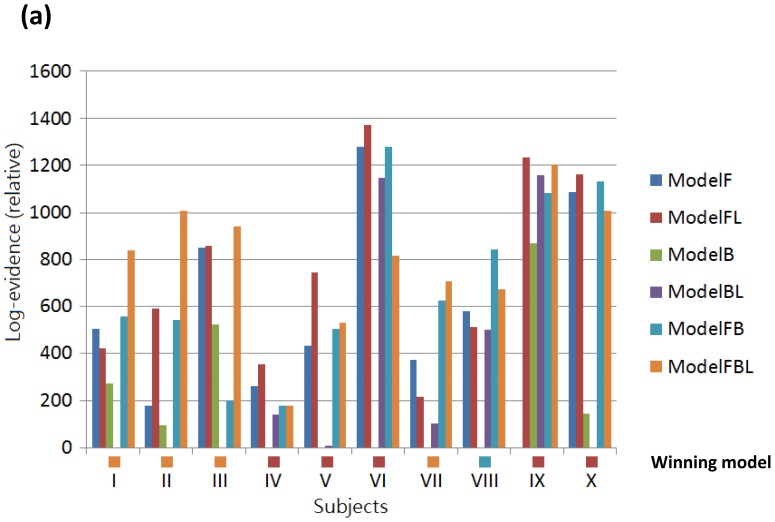
The BMS results of task-dependent modulations under FFX at the single subject level (a) and under RFX at the group level (b).

**Figure 9:**
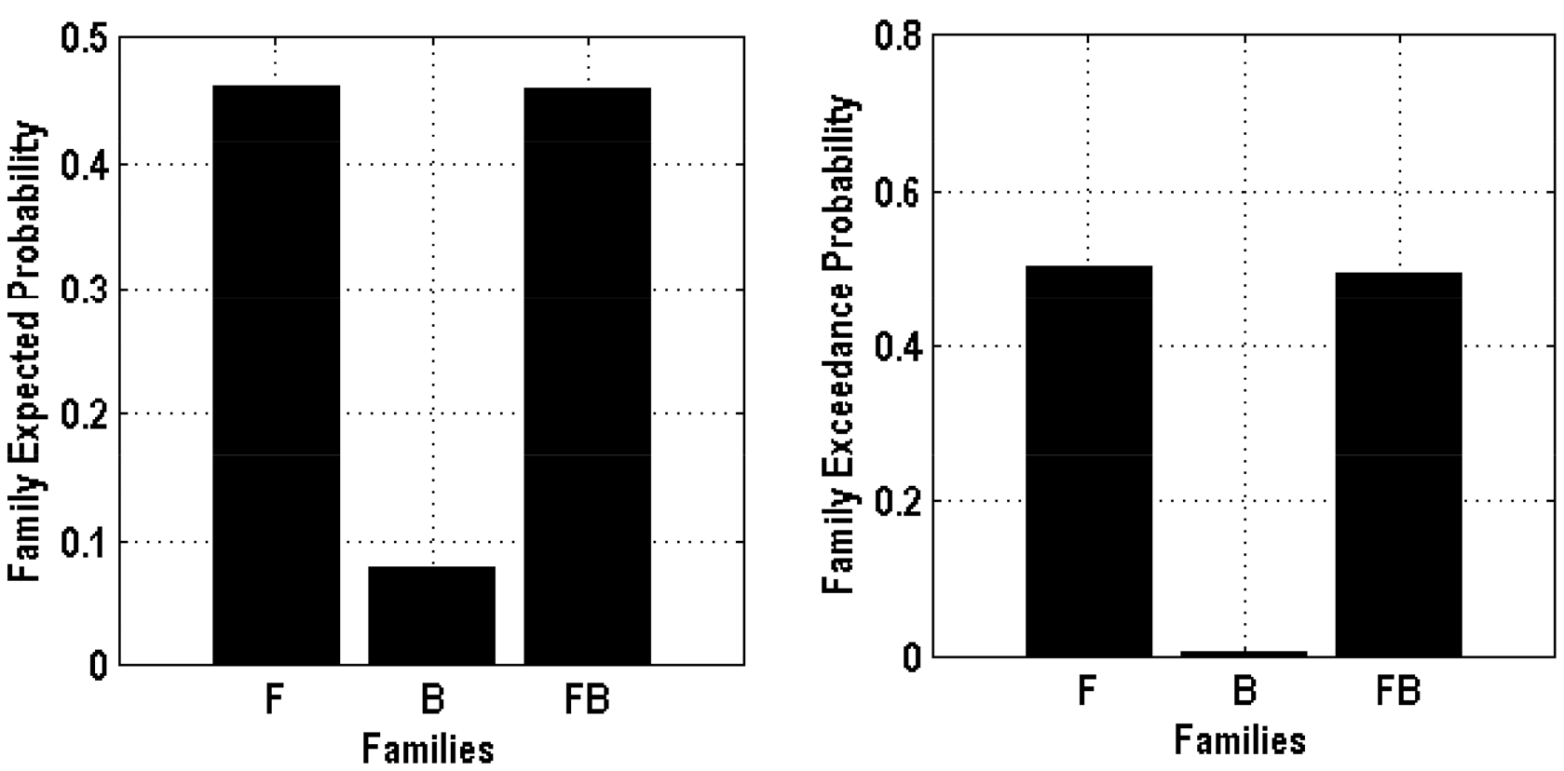
The BMS result of model families.

Six DCMs were inverted for each subject (Figure 1). Figure 5 shows the BMS results at the individual level under the FFX assumption. Seven out of ten subjects have the Model 6 as the best model. At the group level, the BMS results under FFX (Figure 6a) indicated that Model 6 was the winning model without outliers, and the BMS results under the RFX assumption (Figure 6b) confirmed this finding, with a remarkable exceedance probability of 0.7495. Having identified the best model in which the ACC is at the top of this network hierarchy, we then further investigated the task-related modulation mechanism by comparing Model 6 (i.e. FBL model) with 5 derivative models (Figure 7; see **Model Specification for DCM of ERP** section for details). Forward modulation was crucial in this task as the FL and FBL models were optimal for 5 and 4 subjects, respectively (Figure 8a). At the group level, the BMS result under the RFX assumption (Figure 8b) suggested that the FL model is the best (exceedance probability = 0.6934), followed by the FB model (exceedance probability = 0.21). A comparison of the model families (Figure 9) revealed the importance of forward modulation. We observed that the F family was far superior to the B family, and the F and FB families exhibited almost equal exceedance probabilities (0.5005 and 0.4955, respectively). Collectively, forward modulation appeared to be more vital in this passive P300 network, despite the possibility that 2 optimal models could be applied to this task.

### Inference on the Modulatory Effect

The modulation parameter matrix of the winning model from each subject entered the *t* test to verify the inter-individual consistency (i.e. the modulation gain is not equal to 1). We examined the modulation effect among all connections in both FL and FBL model since there was no significant difference between the two models at the individual level. Table 2 listed the statistical results of the modulatory coupling parameters. It can be seen that only forward modulations - from LIPL to LDLPFC and LDLPFC to ACC in the FL model and from LIPL to LDLPFC and RIPL-RDLPFC in the FBL model were were statistically significant.

**Table 2:** The statistical results on the modulatory coupling parameters.

Model FL					
Forward	P-Value	Lateral	P-Value		
LS1-LS2	0.517	LS2-RS2	0.452		
RS1-RS2	0.137	RS2-LS2	0.721		
LS2-LIPL	0.538				
RS2-RIPL	0.876				
**LIPL-LDLPFC**	**0.029** *				
RIPL-RDLPFC	0.071				
**LDLPFC-ACC**	**0.045** *				
RDLPFC-ACC	0.990				
**Model FBL**					
**Forward**	**P-Value**	**Backward**	**P-Value**	**Lateral**	**P-Value**
LS1-LS2	0.978	LS2-LS1	0.050	LS2-RS2	0.921
RS1-RS2	0.488	RS2-RS1	0.841	RS2-LS2	0.298
LS2-LIPL	0.453	LIPL-LS2	0.308		
RS2-RIPL	0.065	RIPL-RS2	0.941		
**LIPL-LDLPFC**	**0.011** *	LDLPFC-LIPL	0.907		
**RIPL-RDLPFC**	**0.010** *	RDLPFC-RIPL	0.689		
LDLPFC-ACC	0.497	ACC-LDLPFC	0.965		
RDLPFC-ACC	0.070	ACC-RDLPFC	0.175		

*P<0.05.

## Discussion

In this study, we have developed a VR-based sensory oddball task to elicit passive P300. The SEPs and P300 were examined to verify the elicitation of passive P300, and the neural network linked to this passive P300 production was identified using DCM and BMS.

### Statistical Analysis of SEPs and P300

To elicit passive P300, we designed a passive sensory paradigm that enables a separation of pure change detection from the response control. The strongest P300 activity was observed at Pz (mean amplitude = 5.14 µV, mean latency = 544 ms), which is in agreement with the findings reported by Duncan et al (2009), Linden (2005), and Polich (2007), thereby supporting the hypothesis that the proposed novel task can reliably elicit P300 activity [2], [3], [32]. It is worth to point out that, despite the time course of the mean SEPs at C3 differed between the 2 conditions (Figure 2), the difference was non-significant. This may be attributed to the constant primary bottom-up sensory input as the subjects held the game controller throughout the experiment. Although the P values of the SEPs increased over time (i.e. *P* = .2 on P50, *P* = .06 on N100, and *P* = .07 on P200), the experimental effect of removing the haptic stimuli was indicated by the elicitation of P300 only, as evidenced by the statistical analysis results (*P*<.05). Similar results have been reported by several previous studies. Akatsuka et al (2007) employed a passive sensory oddball task to examine the effects of stimulus probability, and they observed a non-significant difference of the P50, N80, and P200 peak amplitudes between standard and deviant conditions when the occurrences of the deviant events were 30% and 50% [33]. Bekinschtein et al (2009) used a modified auditory oddball task to probe the consciousness, and found that the only significant difference between the standard and deviant stimuli occurred in the P3b amplitudes [34].

The latency of P300 is thought to reflect, up to some degree, the time needed for processing information while performing the task. The variation in P300 latency is task- and condition-dependent [32]. For instance, P300 latency becomes longer when it is (1) elicited by a visual oddball paradigm than by an auditory one (Bennington and Polich, 1999), (2) elicited by a difficult task than by an easy task [35], (3) elicited under a passive condition than under an active condition [36], or (4) elicited under pathological states such as cognitive impairment [37], [38], [39] or normal aging [40], [41]. Nevertheless, a window of 300–800 ms for each electrode was proved to successfully identify the P300 component under a passive task [36] while a mean tactile P300 latency from 491 to 544 ms at Pz has been reported [41]. Taken together, it is reasonable that we obtained a mean latency of 544 ms of sensory P300 elicited in this passive task.

### Novelty P3, Target P3 and Passive P300

Empirically, there are two subcomponents of P300, target P3/P3b and novelty P3/P3a. Target P3/P3b can be evoked with posterior foci over parietal area when subjects were responding to the target stimulus using a typical oddball paradigm; novelty P3/P3a was identified maximally over frontal electrode when novel rare events were presented using a variant of the oddball paradigm, such as a passive or three-stimulus oddball task [32], [42]. However, a passive paradigm can reliably elicit a comparable central-parietal maximal P3b that was usually obtained under an active condition by a proper task design (e.g. a long enough inter-trial interval in the range of 4–8 s) [36], [43], [44]. This implies that the elicitation of P3a and P3b was influenced largely by the stimulus context [45], [46]. Nevertheless, P3a and P3b can co-occur within the same ERP waveform [42], and they may reflect the output of a widely distributed neural network engaged in attention and memory updating [32], [47], [48]. Hence it seems to be more reasonable to study P3a and P3b as a whole when concerning the generating mechanism.

In this study, the passive P300 was elicited by occasional lack of haptic sensory feedbacks without giving any instruction prior to the experiment. This means that these deviant events were unexpected to the subjects and should be able to elicit the novelty P3a. On the other hand, the long inter-trial interval and the highly task-related infrequent events in this task were intended to produce the typical P3b under a passive condition. Therefore, the passive P300 elicited in this study may comprise of both P3a and P3b, which were mainly manifest over parietal area, and we used a time window from –500 to 900 ms to cover them when modelling the network mechanism in DCM.

### Neuronal Network for Passive P300

In this study, we identified a network comprising the ACC, DLPFC, and IPL, as well as the primary and associative sensory areas for generating passive sensory P300 activity using EEG data. This network has been identified in the previous study [8] for mediating the attentional control using an active somatosensory oddball task and MEG data. fMRI data as well showed the similar results [9], [49]. Brazdil et al (2007) observed a bidirectional network of the ACC, PFC, and IPL for target stimulus processing and they associated this network with two parallel neuronal circuits- frontal (P3a system for top-down attentional control) and parietal circuits (P3b attentional/event encoding system) in target detection [9]. In addition, Asplund et al. (2010) showed that the lateral PFC plays a key role in converging goal-directed and stimulus-driven attention [49]. In other words, the ACC and DLPFC are probably the higher areas providing top-down modulation in the attentional control network. Our findings are in line with these studies specifically because we identified the ACC and DLPFC at top of this network. It is noted that, although the task employed in this study involved a unilateral sensory force input into the right hand, the BMS selects a model with bilateral exogenous inputs to SIs as the wining model. Zhu et al. reported that ipsilateral SI was also activated when using unilateral tactile stimulus [50]. The activity of ipsilateral SI was thought to provide information to SII and the parietal ventral area (PV) for spatiotemporal sensory integration. On the contrast, several studies using unilateral sensory stimuli have reported no significant ipsilateral SI activity though the bilateral SII activations are very evident [6], [51], [52]. The inconsistent results in the previous studies lead to a question whether the ipsilateral SII receives information from ipsilateral SI or contralateral SII or both. In our study, after comparing the models with and without exogenous input to ipsilateral SI (model 6 and 5, respectively) and without inter-hemispheric SII connection (data not shown), we found that information from both ipsilateral SI and contralateral SII are essential inputs to activate the ipsilateral SII.

In summary, our findings are in agreement with those reported by previous studies, implying that a common neural network architecture exists for P300 production, irrespective of the type of stimulus (i.e. sensory, auditory, or visual) or condition (i.e. active or passive).

### Feed-forward Dominating Modulation for a Passive Task

In this study, by omitting haptic stimulus from some of the trials, the P300 was evoked passively through pure change detection, independent of the subjects’ response or attentional control. The BMS results suggested a feed-forward modulatory effect underlying this passive network as evidenced by the exceedance probability values of the FL model under the RFX assumption. This may be explained heuristically; when no response is necessary, no regulation (i.e. the top-down modulation) is required. A study using fMRI data and DCM had shown that the bottom-up stimulus-driven responses, such as surprise processing, engage only the feed-forward connections from the IPL to the ACC and DLPFC, whereas conflict processing modulates only the backward information flow from the ACC and DLPFC to the IPL [8]. In other words, the functional role of forward modulation may indicate the delivery of the sensory information, automatic detection of difference, and the stimulus-driven attentional processes involved in performing the task. This could partly explain our finding of the wining FL model. However, a comparison of the model families under the RFX assumption provides similar evidence for both the F and FB model families, indicating that backward modulation may also play a role in this passive task. From previous studies, it has been shown that backward modulation is involved in attentional control/shifting and motor control [53], [54]. But both the attentional control/shifting and motor control were absent from our experiment as a passive task and can provide only a minor contribution to our model. When taking into account the model parameters, it can be seen that only parameters governing forward modulations are significant. Taken together, this inconsistence may speak to the fact that there is individual variability in response to the experimental manipulation as reflected in two possible models but the most consistent inter-individual modulation effect was observed only in the feed-forward connections.

## Conclusion

In this study, we developed a VR-based sensory oddball task to elicit the passive P300 and investigated the neural network linked to its production by DCM for ERP. The ERP results confirmed that the experimental protocol can reliably elicit passive P300. The DCM results suggested that the passive P300 uses the same parietal-frontal neural network for attentional control, which was identified under an active P300 task. The DCM results also showed that the model with feed-forward modulatory effect wins over the model with backward modulations. To our best knowledge, this is the first study to address the passive P300 network, and these results may provide a reference point for future clinical studies, for example, addressing the network alternations under pathological states of incommunicative patients. However, caution is required when comparing patients’ analytic results with this study. For example, the task presented here is not applicable to incommunicative patients.

## Supporting Information

Appendix S1 **Studies concerning about P300 origins.**
(DOC)

## References

[1] Sutton S, Braren M, Zubin J, John ER (1965) Evoked-potential correlates of stimulus uncertainty. Science 150: 1187–1188. 5852977 10.1126/science.150.3700.1187

[2] Duncan CC, Barry RJ, Connolly JF, Fischer C, Michie PT, et al (2009) Event-related potentials in clinical research: guidelines for eliciting, recording, and quantifying mismatch negativity, P300, and N400. Clin Neurophysiol 120: 1883–1908. 19796989 10.1016/j.clinph.2009.07.045

[3] Linden DE (2005) The p300: where in the brain is it produced and what does it tell us? Neuroscientist 11: 563–576. 16282597 10.1177/1073858405280524

[4] Polich J, Corey-Bloom J (2005) Alzheimer’s disease and P300: review and evaluation of task and modality. Curr Alzheimer Res 2: 515–525. 16375655 10.2174/156720505774932214

[5] Yeung N (2010) Bottom-up influences on voluntary task switching: the elusive homunculus escapes. J Exp Psychol Learn Mem Cogn 36: 348–362. 20192535 10.1037/a0017894

[6] Downar J, Crawley AP, Mikulis DJ, Davis KD (2000) A multimodal cortical network for the detection of changes in the sensory environment. Nat Neurosci 3: 277–283. 10700261 10.1038/72991

[7] Crottaz-Herbette S, Menon V (2006) Where and when the anterior cingulate cortex modulates attentional response: combined fMRI and ERP evidence. J Cogn Neurosci 18: 766–780. 16768376 10.1162/jocn.2006.18.5.766

[8] Huang MX, Lee RR, Miller GA, Thoma RJ, Hanlon FM, et al (2005) A parietal-frontal network studied by somatosensory oddball MEG responses, and its cross-modal consistency. Neuroimage 28: 99–114. 15979344 10.1016/j.neuroimage.2005.05.036

[9] Brazdil M, Mikl M, Marecek R, Krupa P, Rektor I (2007) Effective connectivity in target stimulus processing: a dynamic causal modeling study of visual oddball task. Neuroimage 35: 827–835. 17258910 10.1016/j.neuroimage.2006.12.020

[10] Faugeras F, Rohaut B, Weiss N, Bekinschtein TA, Galanaud D, et al (2011) Probing consciousness with event-related potentials in the vegetative state. Neurology 77: 264–268. 21593438 10.1212/WNL.0b013e3182217ee8 PMC3136052

[11] Perrin F, Schnakers C, Schabus M, Degueldre C, Goldman S, et al (2006) Brain response to one’s own name in vegetative state, minimally conscious state, and locked-in syndrome. Arch Neurol 63: 562–569. 16606770 10.1001/archneur.63.4.562

[12] Rappaport M, McCandless KL, Pond W, Krafft MC (1991) Passive P300 response in traumatic brain injury patients. J Neuropsychiatry Clin Neurosci 3: 180–185. 1821233 10.1176/jnp.3.2.180

[13] Mak JN, Arbel Y, Minett JW, McCane LM, Yuksel B, et al (2011) Optimizing the P300-based brain-computer interface: current status, limitations and future directions. J Neural Eng 8: 025003. 21436525 10.1088/1741-2560/8/2/025003

[14] Wolpaw JR, Birbaumer N, McFarland DJ, Pfurtscheller G, Vaughan TM (2002) Brain-computer interfaces for communication and control. Clin Neurophysiol 113: 767–791. 12048038 10.1016/s1388-2457(02)00057-3

[15] Herbert AM, Gordon GE, McCulloch DL (1998) A ‘passive’ event-related potential? Int J Psychophysiol 28: 11–21. 9506308 10.1016/s0167-8760(97)00070-6

[16] Mertens R, Polich J (1997) P300 from a single-stimulus paradigm: passive versus active tasks and stimulus modality. Electroencephalogr Clin Neurophysiol 104: 488–497. 9402891 10.1016/s0168-5597(97)00041-5

[17] Obuchi C, Harashima T, Shiroma M (2012) Auditory Evoked Potentials under Active and Passive Hearing Conditions in Adult Cochlear Implant Users. Clin Exp Otorhinolaryngol 5 Suppl 1: S6–9. 22701150 10.3342/ceo.2012.5.S1.S6 PMC3369985

[18] John N (2002) Basis and Principles of Virtual Reality in Medical Imaging. In: Caramella D, Bartolozzi, C., editor. 3D Image Processing: Springer Berlin Heidelberg. pp. 279–285.

[19] Laver KE, George S, Thomas S, Deutsch JE, Crotty M (2011) Virtual reality for stroke rehabilitation. Cochrane Database Syst Rev: CD008349. 10.1002/14651858.CD008349.pub3 PMC6465102 25927099

[20] Rizzo A, Schultheis M, Kerns K, Mateer C (2004) Analysis of assets for virtual reality applications in neuropsychology. Neuropsych Rehab 14: 207–223.

[21] Kiebel SJ, Garrido MI, Moran R, Chen CC, Friston KJ (2009) Dynamic causal modeling for EEG and MEG. Hum Brain Mapp 30: 1866–1876. 19360734 10.1002/hbm.20775 PMC6870752

[22] David O, Friston KJ (2003) A neural mass model for MEG/EEG: coupling and neuronal dynamics. Neuroimage 20: 1743–1755. 14642484 10.1016/j.neuroimage.2003.07.015

[23] Duncan CC, Mirsky AF, Lovelace CT, Theodore WH (2009) Assessment of the attention impairment in absence epilepsy: comparison of visual and auditory P300. Int J Psychophysiol 73: 118–122. 19414047 10.1016/j.ijpsycho.2009.03.005 PMC2733346

[24] Chen CC, Kiebel SJ, Kilner JM, Ward NS, Stephan KE, et al (2012) A dynamic causal model for evoked and induced responses. Neuroimage 59: 340–348. 21835251 10.1016/j.neuroimage.2011.07.066 PMC3202632

[25] Montoya P, Sitges C (2006) Affective modulation of somatosensory-evoked potentials elicited by tactile stimulation. Brain Res 1068: 205–212. 16364261 10.1016/j.brainres.2005.11.019

[26] Pullamsetti SS, Berghausen EM, Dabral S, Tretyn A, Butrous E, et al (2012) Role of Src tyrosine kinases in experimental pulmonary hypertension. Arterioscler Thromb Vasc Biol 32: 1354–1365. 22516066 10.1161/ATVBAHA.112.248500

[27] Stephan KE, Penny WD, Daunizeau J, Moran RJ, Friston KJ (2009) Bayesian model selection for group studies. Neuroimage 46: 1004–1017. 19306932 10.1016/j.neuroimage.2009.03.025 PMC2703732

[28] Wang WJ, Hsieh IF, Chen CC (2013) Accelerating Computation of DCM for ERP in MATLAB by External Function Calls to the GPU. PLoS One 8: e66599. 23840507 10.1371/journal.pone.0066599 PMC3694084

[29] Penny WD, Stephan KE, Mechelli A, Friston KJ (2004) Comparing dynamic causal models. Neuroimage 22: 1157–1172. 15219588 10.1016/j.neuroimage.2004.03.026

[30] Stephan KE, Harrison LM, Kiebel SJ, David O, Penny WD, et al (2007) Dynamic causal models of neural system dynamics:current state and future extensions. J Biosci 32: 129–144. 17426386 10.1007/s12038-007-0012-5 PMC2636905

[31] Stephan KE, Penny WD, Moran RJ, den Ouden HE, Daunizeau J, et al (2010) Ten simple rules for dynamic causal modeling. Neuroimage 49: 3099–3109. 19914382 10.1016/j.neuroimage.2009.11.015 PMC2825373

[32] Polich J (2007) Updating P300: an integrative theory of P3a and P3b. Clin Neurophysiol 118: 2128–2148. 17573239 10.1016/j.clinph.2007.04.019 PMC2715154

[33] Akatsuka K, Wasaka T, Nakata H, Kida T, Kakigi R (2007) The effect of stimulus probability on the somatosensory mismatch field. Exp Brain Res 181: 607–614. 17516059 10.1007/s00221-007-0958-4

[34] Bekinschtein TA, Dehaene S, Rohaut B, Tadel F, Cohen L, et al (2009) Neural signature of the conscious processing of auditory regularities. Proc Natl Acad Sci U S A 106: 1672–1677. 19164526 10.1073/pnas.0809667106 PMC2635770

[35] Kok A (2001) On the utility of P3 amplitude as a measure of processing capacity. Psychophysiology 38: 557–577. 11352145 10.1017/s0048577201990559

[36] Jeon YW, Polich J (2001) P3a from a passive visual stimulus task. Clin Neurophysiol 112: 2202–2208. 11738190 10.1016/s1388-2457(01)00663-0

[37] Goodin DS, Aminoff MJ (1992) Evaluation of dementia by event-related potentials. J Clin Neurophysiol 9: 521–525. 1464678 10.1097/00004691-199210000-00006

[38] Lai CL, Lin RT, Liou LM, Liu CK (2010) The role of event-related potentials in cognitive decline in Alzheimer’s disease. Clin Neurophysiol 121: 194–199. 20005164 10.1016/j.clinph.2009.11.001

[39] Picton TW (1992) The P300 wave of the human event-related potential. J Clin Neurophysiol 9: 456–479. 1464675 10.1097/00004691-199210000-00002

[40] Polich J (1996) Meta-analysis of P300 normative aging studies. Psychophysiology 33: 334–353. 8753933 10.1111/j.1469-8986.1996.tb01058.x

[41] Reuter EM, Voelcker-Rehage C, Vieluf S, Winneke AH, Godde B (2013) A parietal-to-frontal shift in the P300 is associated with compensation of tactile discrimination deficits in late middle-aged adults. Psychophysiology. 10.1111/psyp.12037 23517339

[42] Friedman D, Cycowicz YM, Gaeta H (2001) The novelty P3: an event-related brain potential (ERP) sign of the brain’s evaluation of novelty. Neurosci Biobehav Rev 25: 355–373. 11445140 10.1016/s0149-7634(01)00019-7

[43] Polich J, McIsaac HK (1994) Comparison of auditory P300 habituation from active and passive conditions. Int J Psychophysiol 17: 25–34. 7961051 10.1016/0167-8760(94)90052-3

[44] Polich J (1989) P300 from a passive auditory paradigm. Electroencephalogr Clin Neurophysiol 74: 312–320. 2471632 10.1016/0168-5597(89)90061-0

[45] Katayama J, Polich J (1996) P300, probability, and the three-tone paradigm. Electroencephalogr Clin Neurophysiol 100: 555–562. 8980420 10.1016/s0168-5597(96)95171-0

[46] Katayama J, Polich J (1998) Stimulus context determines P3a and P3b. Psychophysiology 35: 23–33. 9499703

[47] Polich J (1988) Bifurcated P300 peaks: P3a and P3b revisited? J Clin Neurophysiol 5: 287–294. 3170723

[48] Wang L, Liu X, Guise KG, Knight RT, Ghajar J, et al (2010) Effective connectivity of the fronto-parietal network during attentional control. J Cogn Neurosci 22: 543–553. 19301995 10.1162/jocn.2009.21210

[49] Asplund CL, Todd JJ, Snyder AP, Marois R (2010) A central role for the lateral prefrontal cortex in goal-directed and stimulus-driven attention. Nat Neurosci 13: 507–512. 20208526 10.1038/nn.2509 PMC2847024

[50] Zhu Z, Disbrow EA, Zumer JM, McGonigle DJ, Nagarajan SS (2007) Spatiotemporal integration of tactile information in human somatosensory cortex. BMC Neurosci 8: 21. 17359544 10.1186/1471-2202-8-21 PMC1838913

[51] Maldjian JA, Gottschalk A, Patel RS, Pincus D, Detre JA, et al (1999) Mapping of secondary somatosensory cortex activation induced by vibrational stimulation: an fMRI study. Brain Res 824: 291–295. 10196461 10.1016/s0006-8993(99)01126-9

[52] Robinson CJ, Burton H (1980) Somatotopographic organization in the second somatosensory area of M. fascicularis. J Comp Neurol 192: 43–67. 7410613 10.1002/cne.901920104

[53] Hopfinger JB, Buonocore MH, Mangun GR (2000) The neural mechanisms of top-down attentional control. Nat Neurosci 3: 284–291. 10700262 10.1038/72999

[54] Narayanan NS, Laubach M (2006) Top-down control of motor cortex ensembles by dorsomedial prefrontal cortex. Neuron 52: 921–931. 17145511 10.1016/j.neuron.2006.10.021 PMC3995137

