# Supplementary material for: Neuronal Correlates of a Virtual-Reality-Based Passive Sensory P300 Network
Source: PLoS One. 2014 Nov 17;9(11):e112228. doi: 10.1371/journal.pone.0112228 (PMC4234463; doi:10.1371/journal.pone.0112228)
Supplement: Appendix S1 — Studies concerning about P300 origins. (DOC) [file pone.0112228.s001.doc]

Appendix SI : Studies concerning about P300 origins

| Area | | EEG/MEG | FMRI | Intracranial Recording |
| --- | --- | --- | --- | --- |
| Frontal | Anterior cingulate cortex | [27][29] | [1][2][3][4][5]  [6][7] [27] [28] | [8][9] |
|  | Orbital frontal cortex |  |  | [10] |
|  | Dorsolateral prefrontal cortex | [11][29] | [1][4][5][6] [12] |  |
|  | Middle frontal gyrus |  | [1][4][5][6][12] |  |
|  | Premotor area | [27] | [27] |  |
| Temporal | Medial temporal lobe | [13][14] |  | [9] |
|  | Temporal-parietal junction | [15] | [28] | [19] |
|  | Hippocampus | [16][17][18] |  |  |
|  | Amygdala |  |  |  |
|  | Superior temporal gyrus | [13][15][20] |  | [19] |
|  | Parahippocampal | [13][16] |  | [19] |
|  | Insular |  | [21] |  |
| Parietal | Posterior parietal cortex | [22] | [21] | [23] |
|  | Inferior parietal cortex | [24][25] [27][29] | [1][2][3][4][5]  [6][7] [21][25][27] | [9] |
|  | Parietal-occipital junction | [20][24] |  | [26] |

[1] M. E. Smith, E. Halgren, M. Sokolik, P. Baudena, A. Musolino, C. Liegeois-Chauvel, and P. Chauvel, “The intracranial topography of the P3 event-related potential elicited during auditory oddball,” *Electroencephalography and Clinical Neurophysiology*, vol. 76, no. 3, pp. 235–248, Sep. 1990.

[2] P. Anderer, R. D. Pascual-Marqui, H. V. Semlitsch, and B. Saletu, “Differential effects of normal aging on sources of standard N1, target N1 and target P300 auditory event-related brain potentials revealed by low resolution electromagnetic tomography (LORETA),” *Electroencephalography and Clinical Neurophysiology/Evoked Potentials Section*, vol. 108, no. 2, pp. 160–174, Mar. 1998.

[3] I. Kiss, R. M. Dashieff, and P. Lordeon, “A parieto-occipital generator for P300: evidence from human intracranial recordings,” *Int. J. Neurosci.*, vol. 49, no. 1–2, pp. 133–139, Nov. 1989.

[4] G. McCarthy, M. Luby, J. Gore, and P. Goldman-Rakic, “Infrequent Events Transiently Activate Human Prefrontal and Parietal Cortex as Measured by Functional MRI,” *J Neurophysiol*, vol. 77, no. 3, pp. 1630–1634, Mar. 1997.

[5] V. Menon, J. M. Ford, K. O. Lim, G. H. Glover, and A. Pfefferbaum, “Combined event-related fMRI and EEG evidence for temporal-parietal cortex activation during target detection,” *Neuroreport*, vol. 8, no. 14, pp. 3029–3037.

[6] D. E. J. Linden, D. Prvulovic, E. Formisano, M. Völlinger, F. E. Zanella, R. Goebel, and T. Dierks, “The Functional Neuroanatomy of Target Detection: An fMRI Study of Visual and Auditory Oddball Tasks,” *Cereb. Cortex*, vol. 9, no. 8, pp. 815–823, Dec. 1999.

[7] T. Yoshiura, J. Zhong, D. K. Shibata, W. E. Kwok, D. A. Shrier, and Y. Numaguchi, “Functional MRI study of auditory and visual oddball tasks,” *Neuroreport*, vol. 10, no. 8, pp. 1683–1688.

[8] J. Polich and A. Kok, “Cognitive and biological determinants of P300: an integrative review,” *Biological Psychology*, vol. 41, no. 2, pp. 103–146, Oct. 1995.

[9] J. Wang, K.-I. Hiramatsu, H. Hokama, H. Miyazato, and C. Ogura, “Abnormalities of auditory P300 cortical current density in patients with schizophrenia using high density recording,” *International Journal of Psychophysiology*, vol. 47, no. 3, pp. 243–253, Mar. 2003.

[10] D. E. J. Linden, “The P300: Where in the Brain Is It Produced and What Does It Tell Us?,” *Neuroscientist*, vol. 11, no. 6, pp. 563–576, Dec. 2005.

[11] M. Huang, C. . Aine, S. Supek, E. Best, D. Ranken, and E. . Flynn, “Multi-start downhill simplex method for spatio-temporal source localization in magnetoencephalography,” *Electroencephalography and Clinical Neurophysiology/Evoked Potentials Section*, vol. 108, no. 1, pp. 32–44, Jan. 1998.

[12] V. P. Clark, S. Fannon, S. Lai, R. Benson, and L. Bauer, “Responses to Rare Visual Target and Distractor Stimuli Using Event-Related fMRI,” *J Neurophysiol*, vol. 83, no. 5, pp. 3133–3139, May 2000.

[13] C. Wang, I. Ulbert, D. L. Schomer, K. Marinkovic, and E. Halgren, “Responses of Human Anterior Cingulate Cortex Microdomains to Error Detection, Conflict Monitoring, Stimulus-Response Mapping, Familiarity, and Orienting,” *J. Neurosci.*, vol. 25, no. 3, pp. 604–613, Jan. 2005.

[14] P. Baudena, E. Halgren, G. Heit, and J. M. Clarke, “Intracerebral potentials to rare target and distractor auditory and visual stimuli. III. Frontal cortex,” *Electroencephalography and Clinical Neurophysiology*, vol. 94, no. 4, pp. 251–264, Apr. 1995.

[15] V. Molina, J. Sanz, F. Muñoz, P. Casado, J. A. Hinojosa, F. Sarramea, and M. Martín-Loeches, “Dorsolateral prefrontal cortex contribution to abnormalities of the P300 component of the event-related potential in schizophrenia,” *Psychiatry Research: Neuroimaging*, vol. 140, no. 1, pp. 17–26, Oct. 2005.

[16] I. M. Tarkka, D. S. Stokić, L. F. H. Basile, and A. C. Papanicolaou, “Electric source localization of the auditory P300 agrees with magnetic source localization,” *Electroencephalography and Clinical Neurophysiology/Evoked Potentials Section*, vol. 96, no. 6, pp. 538–545, Nov. 1995.

[17] B. F. O’Donnell, R. A. Cohen, H. Hokama, B. N. Cuffin, C. Lippa, M. E. Shenton, and D. A. Drachman, “Electrical source analysis of auditory ERPs in medial temporal lobe amnestic syndrome,” *Electroencephalography and Clinical Neurophysiology*, vol. 87, no. 6, pp. 394–402, Dec. 1993.

[18] K. A. Kiehl and P. F. Liddle, “An event-related functional magnetic resonance imaging study of an auditory oddball task in schizophrenia,” *Schizophrenia Research*, vol. 48, no. 2–3, pp. 159–171, Mar. 2001.

[19] R. T. Knight, D. Scabini, D. L. Woods, and C. C. Clayworth, “Contributions of temporal-parietal junction to the human auditory P3,” *Brain Research*, vol. 502, no. 1, pp. 109–116, Nov. 1989.

[20] I. M. Tarkka, S. Micheloyannis, and D. . Stokić, “Generators for human P300 elicited by somatosensory stimuli using multiple dipole source analysis,” *Neuroscience*, vol. 75, no. 1, pp. 275–287, Sep. 1996.

[21] J. L. Kenemans and S. Kähkönen, “How Human Electrophysiology Informs Psychopharmacology: from Bottom-up Driven Processing to Top-Down Control,” *Neuropsychopharmacology*, vol. 36, no. 1, pp. 26–51, Oct. 2010.

[22] E. Halgren, N. K. Squires, C. L. Wilson, J. W. Rohrbaugh, T. L. Babb, and P. H. Crandall, “Endogenous Potentials Generated in the Human Hippocampal Formation and Amygdala by Infrequent Events,” *Science*, vol. 210, no. 4471, pp. 803–805, Nov. 1980.

[23] E. Ludowig, C. G. Bien, C. E. Elger, and T. Rosburg, “Two P300 generators in the hippocampal formation,” *Hippocampus*, vol. 20, no. 1, pp. 186–195, May 2009.

[24] E. Halgren, P. Baudena, J. M. Clarke, G. Heit, C. Liégeois, P. Chauvel, and A. Musolino, “Intracerebral potentials to rare target and distractor auditory and visual stimuli. I. Superior temporal plane and parietal lobe,” *Electroencephalography and Clinical Neurophysiology*, vol. 94, no. 3, pp. 191–220, Mar. 1995.

[25] B. A. Ardekani, S. J. Choi, G.-A. Hossein-Zadeh, B. Porjesz, J. L. Tanabe, K. O. Lim, R. Bilder, J. A. Helpern, and H. Begleiter, “Functional magnetic resonance imaging of brain activity in the visual oddball task,” *Cognitive Brain Research*, vol. 14, no. 3, pp. 347–356, Nov. 2002.

[26] B. He, J. Lian, K. M. Spencer, J. Dien, and E. Donchin, “A cortical potential imaging analysis of the P300 and Novelty P3 components,” *Human Brain Mapping*, vol. 12, no. 2, pp. 120–130, Jan. 2001.

[27] S. Crottaz-Herbette, V. Menon, "Where and when the anterior cingulate cortex modulates attentional response: combined fMRI and ERP evidence". J Cogn Neurosci 18, 766-780. 2006.

[28] J. Downar, A. P. Crawley, D. J. Mikulis, K. D. Davis, "A multimodal cortical network for the detection of changes in the sensory environment". Nat Neurosci 3, 277-283, 2000

[29] M. X. Huang, R. R. Lee, G. A. Miller, R. J. Thoma, F. M. Hanlon, K. M. Paulson, K. Martin, D. Harrington, M. Weisend, J. Edgar, J. Canive, "A parietal-frontal network studied by somatosensory oddball MEG responses, and its cross-modal consistency". Neuroimage 28, 99-114, 2005
